# Supplementary material for: Effects of Space Flight on Mouse Liver versus Kidney: Gene Pathway Analyses
Source: Int J Mol Sci. 2018 Dec 18;19(12):4106. doi: 10.3390/ijms19124106 (PMC6321533; doi:10.3390/ijms19124106)
Supplement: Supplementary file 1 [file ijms-19-04106-s001.zip › Supplementary Figure S2.pdf]

Mouse Kidney Overexpression Specific Cluster

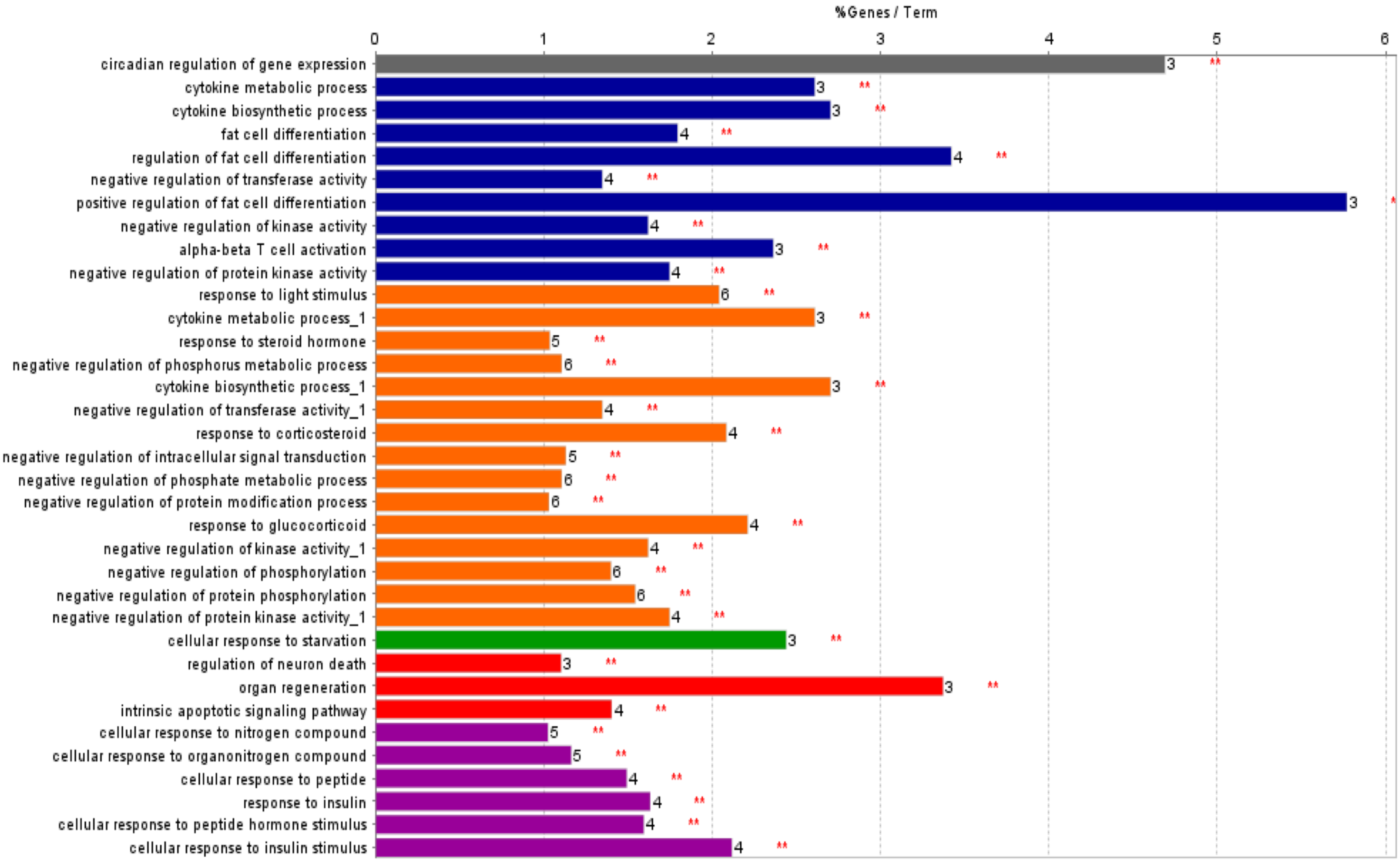

Supplemental Figure 2. For each Gene Ontology (GO) biological process shown in Figure 4, the length of the bar in the graph above indicates the percent of the genes in that GO term. The number to the right of the bar indicates the percentage of the total number of genes in that GO term. Bar colors are matched to the cluster colors in Fig 4.
